# Rat tail static compression model mimics extracellular matrix metabolic imbalances of matrix metalloproteinases, aggrecanases, and tissue inhibitors of metalloproteinases in intervertebral disc degeneration

**DOI:** 10.1186/ar3764

**Published:** 2012-03-06

**Authors:** Takashi Yurube, Toru Takada, Teppei Suzuki, Kenichiro Kakutani, Koichiro Maeno, Minoru Doita, Masahiro Kurosaka, Kotaro Nishida

**Affiliations:** 1 Department of Orthopaedic Surgery, Kobe University Graduate School of Medicine, Kobe, Japan

## Abstract

**Introduction:**

The longitudinal degradation mechanism of extracellular matrix (ECM) in the interbertebral disc remains unclear. Our objective was to elucidate catabolic and anabolic gene expression profiles and their balances in intervertebral disc degeneration using a static compression model.

**Methods:**

Forty-eight 12-week-old male Sprague-Dawley rat tails were instrumented with an Ilizarov-type device with springs and loaded statically at 1.3 MPa for up to 56 days. Experimental loaded and distal-unloaded control discs were harvested and analyzed by real-time reverse transcription-polymerase chain reaction (PCR) messenger RNA quantification for catabolic genes [*matrix metalloproteinase *(*MMP*)*-1a*, *MMP-2*, *MMP-3*, *MMP-7*, *MMP-9*, *MMP-13*, *a disintegrin and metalloproteinase with thrombospondin motifs *(*ADAMTS*)*-4*, and *ADAMTS-5*], anti-catabolic genes [*tissue inhibitor of metalloproteinases *(*TIMP*)*-1*, *TIMP-2*, and *TIMP-3*], ECM genes [*aggrecan-1*, *collagen type 1-α1*, and *collagen type 2-α1*], and pro-inflammatory cytokine genes [*tumor necrosis factor *(*TNF*)*-α*, *interleukin *(*IL*)*-1α*, *IL-1β*, and *IL-6*]. Immunohistochemistry for MMP-3, ADAMTS-4, ADAMTS-5, TIMP-1, TIMP-2, and TIMP-3 was performed to assess their protein expression level and distribution. The presence of MMP- and aggrecanase-cleaved aggrecan neoepitopes was similarly investigated to evaluate aggrecanolytic activity.

**Results:**

Quantitative PCR demonstrated up-regulation of all *MMPs *and *ADAMTS-4 *but not *ADAMTS-5. TIMP-1 *and *TIMP-2 *were almost unchanged while *TIMP-3 *was down-regulated. Down-regulation of *aggrecan-1 *and *collagen type 2-α1 *and up-regulation of *collagen type 1-α1 *were observed. Despite *TNF-α *elevation, *ILs *developed little to no up-regulation. Immunohistochemistry showed, in the nucleus pulposus, the percentage of immunopositive cells of MMP-cleaved aggrecan neoepitope increased from 7 through 56 days with increased MMP-3 and decreased TIMP-1 and TIMP-2 immunopositivity. The percentage of immunopositive cells of aggrecanase-cleaved aggrecan neoepitope increased at 7 and 28 days only with decreased TIMP-3 immunopositivity. In the annulus fibrosus, MMP-cleaved aggrecan neoepitope presented much the same expression pattern. Aggrecanase-cleaved aggrecan neoepitope increased at 7 and 28 days only with increased ADAMTS-4 and ADAMTS-5 immunopositivity.

**Conclusions:**

This rat tail sustained static compression model mimics ECM metabolic imbalances of MMPs, aggrecanases, and TIMPs in human degenerative discs. A dominant imbalance of MMP-3/TIMP-1 and TIMP-2 relative to ADAMTS-4 and ADAMTS-5/TIMP-3 signifies an advanced stage of intervertebral disc degeneration.

## Introduction

Low back pain is a global health problem due to its high prevalence and high socioeconomic burden. It affects 70 to 85% of the population during a lifetime, 15 to 45% in a year, and 12 to 30% at any point, and accounts for approximately 13% of sickness absences [1]. Although the cause of low back pain is multifactorial, intervertebral disc degeneration is implicated in more than half of the cases [2].

The intervertebral disc has a complex structure with the nucleus pulposus (NP) encapsulated by endplates and the annulus fibrosus (AF). Intervertebral disc degeneration is biochemically characterized by extracellular matrix (ECM) degradation [3-5]. ECM consists primarily of proteoglycans -- principally aggrecan -- and collagens -- mainly type 2 in the NP and type 1 in the AF [6]. ECM metabolism is regulated by the balance between degradative enzymes, matrix metalloproteinases (MMPs) and aggrecanases, and their natural inhibitors, tissue inhibitors of metalloproteinases (TIMPs) [7,8]. Aggrecanases are identified as members of a disintegrin and metalloproteinase with thrombospondin motifs (ADAMTS) family [7]. Imbalances of MMPs, ADAMTSs, and TIMPs significantly correlate with cartilage ECM metabolism in patients with osteoarthritis and rheumatoid arthritis [9-11]. In degenerated disc tissue, modified expressions of MMPs, ADAMTSs, and TIMPs have also been detected [12-19]. However, balances of these enzymes and their practical significance in intervertebral disc degeneration remain unclear.

Studying disc degeneration is difficult because of the challenge of reproducing the variety of etiological aspects of the degenerative process: ECM degradation, inflammation, nutrient loss, cell senescence, and apoptotic cell death [20]. Systematic analysis of these etiologies using human specimens is impractical; therefore, reliable animal models of disc degeneration are required.

Rodent tails are popular to assess disc degeneration because of easy accessibility with minimal damage to surrounding tissues and minimal interference with normal physiological functions [21]. Rodents keep notochordal cells in the disc NP throughout their lifetime [21] whereas humans lose them at young ages in somatic development, when discs begin to show first signs of degeneration [22]. Recent evidence has suggested that the change of NP cell phenotype from notochordal to chondrocyte-like plays a significant role in the initiation of disc degeneration [23,24]. Thus, understanding rodent disc degeneration provides an interpretation of the pathogenesis of human disc degeneration.

Many methods to induce degeneration are proposed; mechanical loading provokes chronic degenerative responses unlike annular puncture which provides reliable responses to acute injury [21]. Mounting evidence has revealed that dynamic compression stimulates anabolism whereas static compression accelerates catabolism [25-27]. Static compression induces histomorphological degeneration [28-30], cell apoptosis [28-32], and altered content of proteoglycans [25,28,29,33] and collagens [28,29,34,35]. Static compression thus has the potential to reproduce disc degeneration via cell apoptosis and ECM degradation; this conveys its primary advantage for longitudinal investigation of the degenerative mechanism compared with dynamic compression [21,36]. ECM metabolism under static compression has been partially explained by activation of MMP-2 [37] and up-regulation of MMP-13 and TIMP-1 [34,35]. The authors have previously reported that *in vivo *sustained static compression leads to progressive and prolonged up-regulation of MMP-3 with the progression of radiological and histomorphological degeneration [38]. However, comprehensive degeneration-related gene expression including MMP, ADAMTS, and TIMP balances has not been profiled. Their ECM degradation potential has not been the focus of investigation. Therefore, longitudinal gene-quantification studies using the static compression model still need to be conducted.

The objectives of this study were to clarify catabolic and anabolic gene expression profiles and to elucidate balances of MMPs, aggrecanases, and TIMPs in ECM metabolism of intervertebral disc degeneration.

## Materials and methods

All animal procedures were performed under the approval and guidance of the Animal Care and Use Committee at Kobe University Graduate School of Medicine.

### Animals and surgical procedure

**Figure 1:**
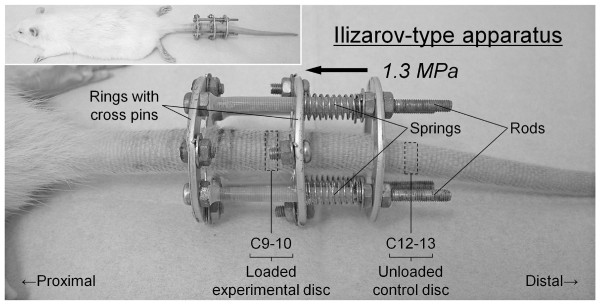
**Whole and close-up view of rat tail instrumented with an Ilizarov-type loading device**.

Forty-eight 12-week-old male Sprague-Dawley rats (CLEA Japan, Tokyo, Japan), ranging in weight from 452 to 509 g, were used. Rats are reported to reach approximately 90% of skeletal maturity 12 weeks after birth [39]. Rat tails were affixed with an Ilizarov-type apparatus with springs between the 8^th ^and 10^th ^coccygeal (C) vertebrae as described in our previous paper (Figure 1) [38]. This loading system was similar to that of Iatridis and colleagues [33]. Under intraperitoneal anesthesia, two-cross 0.7 mm diameter Kirschner wires were inserted percutaneously into each vertebral body perpendicular to the tail's axis and attached to aluminum rings. Rings were connected longitudinally with four threaded rods. Four 0.50 N/mm calibrated springs were installed over each rod. After instrumentation, axial stress was loaded from the distal side to produce a calculated compressive pressure of 1.3 MPa. This pressure, corresponding closely to the disc loading force produced by lifting a moderate weight in the human lumbar spine, was shown to induce morphological and biochemical disc degeneration with cell apoptosis by Lotz and colleagues [28,31].

Following surgery, rats were randomly loaded for 0 (sham), 7, 28, or 56 days and euthanized; the data did not consist of repeated measurements over time points, but of single measurements in each time point. Rat tails with the compressive apparatus unloaded for up to 56 days were used as the sham group. In 24 rats, C9-10, the distal loaded disc, and C12-13, the unloaded internal control disc [38], were harvested for messenger RNA (mRNA) quantification following radiographic and magnetic resonance imaging (MRI) assessments (*n *= 6/time point). To exclude potential level effects [40], those discs in the additional 24 rats were harvested for histomorphological and immunohistochemical assessments (*n *= 6/time point). Radiological, histomorphological, and cell population data were presented previously [38,41]. No obvious change in adjacent disc levels of the Ilizarov-type device over 56 days was confirmed biochemically [33] and radiologically [38].

### RNA extraction and reverse transcription

Loaded and unloaded discs were immediately dissected using a scalpel after euthanasia. NP tissue was collected using a curette, pulverized under liquid nitrogen, and total RNA was isolated using RNeasy Mini Kit (Qiagen, Hilden, Germany). Then 0.1 μg RNA was reverse-transcribed in the presence of RT^2 ^First Strand Kit (SABiosciences, Frederick, MD, USA) including oligo d(T) primer and random hexamers.

### Quantitative real-time reverse transcription-polymerase chain reaction

#### Catabolic genes

Relative expression levels of mRNA encoding rat *MMP-1a *(GenBank: NM_001134530), *MMP-2 *(GenBank: NM_031054), *MMP-3 *(GenBank: NM_133523), *MMP-7 *(GenBank: NM_012864), *MMP-9 *(GenBank: NM_031055), *MMP-13 *(GenBank: NM_133530), *ADAMTS-4 *(GenBank: NM_023959), and *ADAMTS-5 *(GenBank: NM_198761) were calculated by real-time reverse transcription (RT)-polymerase chain reaction (PCR) using ABI Prism 7500 sequence detection system (Applied Biosystems, Foster City, CA, USA). *MMP-1a *was predicted to encode a rat homolog of human MMP-1 [42] because rodent species appear to lack MMP-1 [43]. *Glyceraldehyde 3-phosphate dehydrogenase *(*GAPDH*) (GenBank: NM_017008) mRNA expression was measured as an endogenous control. Good feasibility of *GAPDH *was confirmed in our previous experiment using this rat tail model [41]. We used a custom-made RT^2 ^Profiler PCR Array (SABiosciences, Frederick, MD, USA), which consisted of a set of SYBR green fluorescent dye and multiple pre-designed primers with an arrangement to analyze multiple target gene mRNA expressions of experimental and control samples simultaneously. We separately used primers for *MMP-3 *(Takarabio, Shiga, Japan) in order to amplify a specific sequence previously published: sense 5'-TGG ACC AGG GAC CAA TGG A-3' and anti-sense 5'-GGC CAA GTT CAT GAG CAG CA-3' [38]. The use of the customized PCR Array system made it possible to precisely measure multiple gene alterations in the same rat samples under the same experimental conditions. The measurement was performed in duplicate. The mRNA expression of each enzyme in the C9-10 loaded disc was converted to a relative number representing the amount of mRNA compared with the C12-13 unloaded disc using the 2^-ΔΔCt ^method [44]. Difference in threshold cycles for target gene and reference gene was calculated as ΔCt = Ct_target gene _- Ct_GAPDH_. Difference in threshold cycles for target sample and reference sample was calculated as ΔΔCt = ΔCt_C9-10 _- ΔCt_C12-13_. Finally, the mRNA expression fold change of target gene in the loaded relative to unloaded disc was calculated as 2^-ΔΔCt^.

#### Anti-catabolic genes

*TIMP-1 *(GenBank: NM_053819), *TIMP-2 *(GenBank: NM_021989), and *TIMP-3 *(GenBank: NM_012886) mRNA expression levels were quantified following the same PCR array procedure.

#### Extracellular matrix genes

*Aggrecan-1 *(GenBank: NM_022190), *collagen type 1-α1 *(GenBank: NM_053304), and *collagen type 2-α1 *(GenBank: NM_012929) mRNA expression levels were assessed using PCR array.

#### Pro-inflammatory cytokines genes

*TNF-α *(GenBank: NM_012675), *IL-1α *(GenBank: NM_017019), *IL-1β *(GenBank: NM_031512), and *IL-6 *(GenBank: NM_012589) mRNA expression levels were evaluated with PCR array.

### Paraffin-embedded disc tissue preparation

Loaded and unloaded discs were excised, fixed in 4% paraformaldehyde, decalcified in 10% ethylenediaminetetraacetic acid, embedded in paraffin, sectioned from the mid-sagittal plane at 5-μm thickness, and prepared for immunohistochemical analysis.

### Immunohistochemistry

#### Catabolic and anti-catabolic genes

Immunohistochemical staining was performed to determine the level and distribution of protein expression of MMP-3, ADAMTS-4, ADAMTS-5, TIMP-1, TIMP-2, and TIMP-3. Sections were incubated with 1:75 diluted goat-polyclonal anti-MMP-3 (sc-6839; Santa Cruz Biotechnology, Santa Cruz, CA, USA), 1:100 diluted rabbit-polyclonal anti-ADAMTS-4 (SP4703P; Acris Antibodies, Herford, Germany), 1:20 diluted rabbit-polyclonal anti-ADAMTS-5 (sc-83186; Santa Cruz Biotechnology, Santa Cruz, CA, USA), 1:50 diluted rabbit-polyclonal anti-TIMP-1 (ab78703; Abcam, Cambridge, UK), 1:50 diluted rabbit-polyclonal anti-TIMP-2 (sc-5539; Santa Cruz Biotechnology, Santa Cruz, CA, USA), or 1:20 diluted mouse-monoclonal anti-TIMP-3 (ab49670; Abcam, Cambridge, UK) at 4°C overnight, and subsequently treated at room temperature for 30 minutes with a peroxidase-labeled anti-goat, anti-mouse, or anti-rabbit antibody (Nichirei Bioscience, Tokyo, Japan). The signal, a brown reaction product, was developed using peroxidase substrate 3,3'-diaminobenzide and counterstained with hematoxylin. Parallel sections treated with goat, mouse, or rabbit normal serum (Dako, Glostrup, Denmark) at equal protein concentrations were used as negative controls. The number of immunopositive cells of each NP and AF dissected on the center was counted in five random high-power fields (×400) using BZ-9000 microscope and analysis software (Keyence, Osaka, Japan); positive staining was expressed as a percentage of immunopositive cells to total cell population measured by counting the nuclei.

### Aggrecanolytic activity

Immunohistochemistry using cleavage site-specific antibodies for aggrecan was performed to detect aggrecan core protein fragments generated by MMP and aggrecanase activity. After deglycosylation of the aggrecan core proteins using chondroitinase ABC (Sigma-Aldrich, St. Louis, MO, USA) and keratanases (Seikagaku Biobusiness, Tokyo, Japan), sections were treated at 4°C overnight with 1:20 diluted mouse-monoclonal antibody BC-14 (NB110-6852; Novus Biologicals, Littleton, CO, USA), which recognized the N-terminal neoepitope sequence of ^342^FFGVG generated by MMP cleavage in the intergrobular domain of aggrecan, or 1:100 diluted mouse-monoclonal antibody BC-3 (NB600-504; Novus Biologicals, Littleton, CO, USA), which recognized the N-terminal neoepitope sequence of ^374^ARGSV generated by aggrecanase cleavage. The secondary antibody was a peroxidase-labeled anti-mouse antibody (Nichirei Bioscience, Tokyo, Japan) used at room temperature for 30 minutes. Brown color-development, counterstaining, cell-counting, and negative control preparation were performed as described above.

### Statistical analysis

Two-way mixed-design analysis of variance (ANOVA) with the Turkey-Kramer post-hoc test was used to assess changes of mRNA level for the effects of disc level (loaded and unloaded: within-subjects) and time (0, 7, 28, and 56 days: between-subjects). Two-way ANOVA with the Turkey-Kramer post-hoc test was applied to evaluate changes of percentage of immunopositive cells similarly (both factors: between-subjects). Data analyses were performed using PASW Statistics 18 (SPSS, Chicago, IL, USA). Statistical significance was accepted at *P *< 0.05. All values are expressed as mean ± standard deviation.

## Results

All animals tolerated surgery well and gained body weight throughout the experiment: 455 to 526 g at 7 days, 497 to 563 g at 28 days, and 543 to 614 g at 56 days. All springs maintained their compressive length and fully recovered immediately after release, indicating sustained axial loading. No signs of infection, skin necrosis, neurological problems, or instrument failure were observed.

### Quantitative real-time reverse transcription-polymerase chain reaction outcomes

For application of the 2^-ΔΔCt ^method, the efficiencies of all measured gene amplifications were first examined using diluted samples and confirmed to be appropriately equal. In addition, no amplification of primer dimers and other non-specific products were detected.

### Catabolic genes

**Figure 2:**
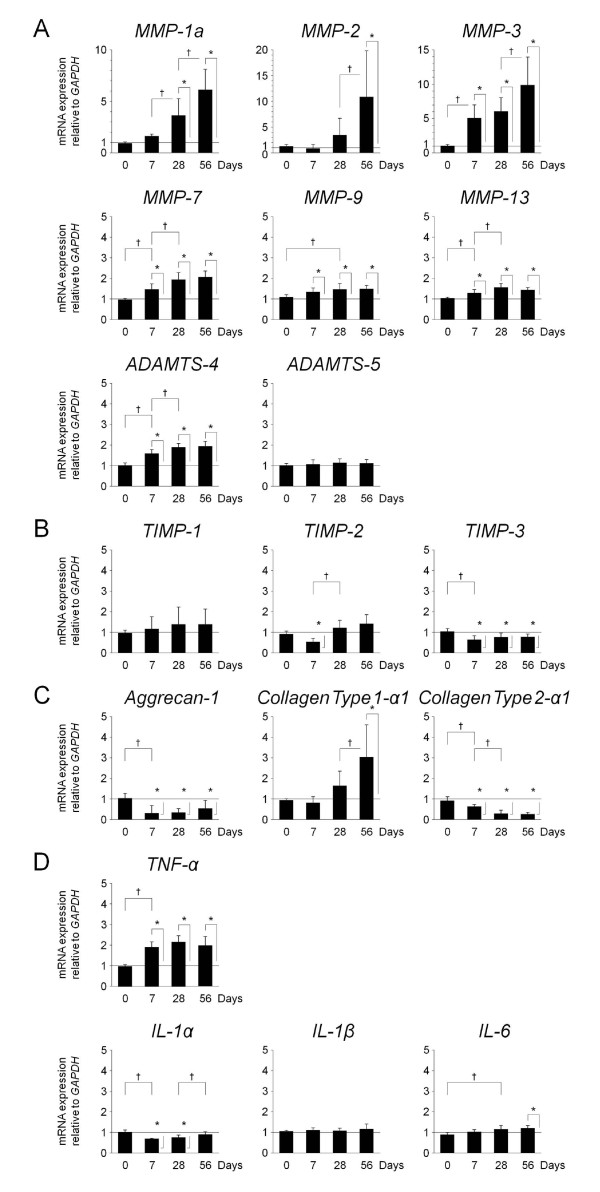
**Real-time reverse transcription-polymerase chain reaction gene expression profile**. The messenger RNA (mRNA) expression of target gene normalized to *glyceraldehyde 3-phosphate dehydrogenase *(*GAPDH*) is represented by fold change in the loaded relative to unloaded disc (control value = 1). *P < 0.05 when compared between loaded and unloaded conditions. ^†^*P *< 0.05 when compared between different time points. **(a) **Relative mRNA expression at 0, 7, 28, and 56 days of: catabolic *matrix metalloproteinase *(*MMP*)*-1a *= 0.9, 1.6, 3.6, 6.1, respectively; *MMP-2 *= 1.2, 0.8, 3.4, 10.8, respectively; *MMP-3 *= 1.0, 5.0, 6.0, 9.8, respectively; *MMP-7 *= 1.0, 1.4, 1.9, 2.0, respectively; *MMP-9 *= 1.1, 1.3, 1.5, 1.5, respectively; *MMP-13 *= 1.0, 1.3, 1.5, 1.4, respectively; *a disintegrin and metalloproteinase with thrombospondin motifs *(*ADAMTS*)*-4 *= 1.0, 1.6, 1.9, 1.9, respectively; *ADAMTS-5 *= 1.0, 1.1, 1.1, 1.1, respectively. **(b) **Relative mRNA expression at 0, 7, 28, and 56 days of: anti-catabolic *tissue inhibitor of metalloproteinases *(*TIMP*)*-1 *= 1.0, 1.2, 1.4, 1.4, respectively; *TIMP-2 *= 0.9, 0.5, 1.2, 1.4, respectively; *TIMP-3 *= 1.0, 0.6, 0.7, 0.8, respectively. **(c) **Relative mRNA expression at 0, 7, 28, and 56 days of: extracellular matrix *aggrecan-1 *= 1.0, 0.3, 0.3, 0.5, respectively; *collagen type 1-α1 *= 0.9, 0.8, 1.6, 3.0, respectively; *collagen type 2-α1 *= 0.9, 0.6, 0.3, 0.2, respectively. **(d) **Relative mRNA expression at 0, 7, 28, and 56 days of: pro-inflammatory cytokine *tumor necrosis factor *(*TNF*)*-α *= 1.0, 1.9, 2.2, 2.0, respectively; *interleukin *(*IL*)*-1α *= 1.0, 0.7, 0.8, 0.9, respectively; *IL-1β *= 1.1, 1.1, 1.1, 1.2, respectively; *IL-6 *= 0.9, 1.0, 1.2, 1.2, respectively.

High-throughput mRNA quantification of the loaded relative to unloaded disc demonstrated significant up-regulation of all examined catabolic genes except *ADAMTS-5 *(Figure 2a). *MMP-3*, *MMP-7*, *MMP-9*, and *MMP-13 *showed significant up-regulation from seven days of loading, *MMP-1a *from 28 days, and *MMP-2 *from 56 days (*P *< 0.05). Furthermore, *MMP *up-regulation significantly progressed over time with compression (*P *< 0.05). In aggrecanases, *ADAMTS-4 *exhibited similar significant up-regulation from seven days (*P *< 0.05) while *ADAMTS-5 *underwent no significant up-regulation throughout the study duration. The increasing tendency was more remarkable in *MMP-1a*, *MMP-2*, and *MMP-3 *than *MMP-7*, *MMP-9*, *MMP-13*, and *ADAMTS-4*. In the mRNA expression of these enzymes at seven days, *MMP-3 *showed the most notable increase.

#### Anti-catabolic genes

All *TIMPs *demonstrated no obvious up-regulation during the loading period (Figure 2b). *TIMP-1 *exhibited no significant change but did show an increasing trend toward up-regulation. *TIMP-2 *showed significant down-regulation at seven days (*P *< 0.05) and it recovered at 28 days (*P *< 0.05). Meanwhile, *TIMP-3 *was significantly and continuously down-regulated from seven days of loading (*P *< 0.05).

#### Extracellular matrix genes

*Aggrecan-1 *and *collagen type 2-α1 *were significantly down-regulated from seven days (*P *< 0.05) whereas *collagen type 1-α1 *was up-regulated with significance at 56 days (*P *< 0.05) (Figure 2c).

#### Pro-inflammatory cytokine genes

*TNF-α *showed significant up-regulation at seven days (*P *< 0.05) and subsequently maintained high expression levels through 56 days (Figure 2d). However, *IL *expression demonstrated little to no elevation; significant down-regulation of *IL-1α *at 7 and 28 days (*P *< 0.05), no significant change of *IL-1β *throughout, and significant up-regulation of *IL-6 *only at 56 days (*P *< 0.05) were observed (Figure 2d).

### Immunohistochemical outcomes

The number of disc cells progressively decreased with compression. In the NP, while large vacuolated notochordal cells were frequently observed at 0 day but apparently disappeared from seven days, smaller round chondrocyte-like cells clustered and collapsed elliptically but were found throughout the study. In the AF, evenly distributed fibroblast-like cells were observed at 0 day but subsequently decreased and larger round chondrocyte-like cells appeared.

#### Catabolic and anti-catabolic genes

**Figure 3:**
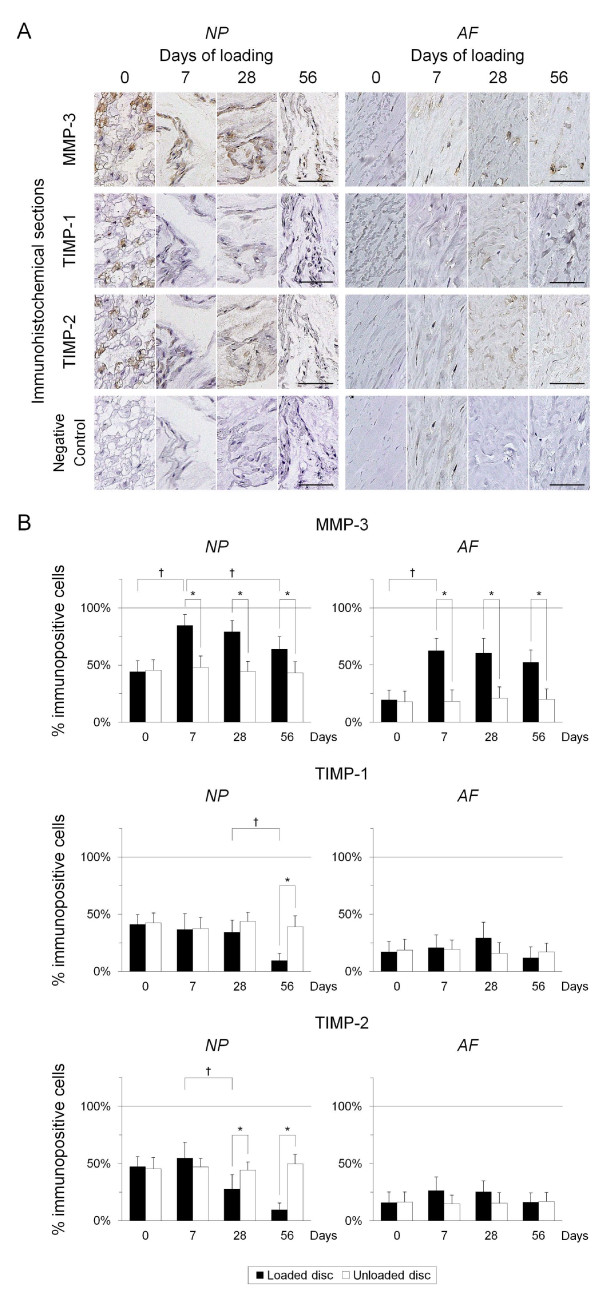
**Immunohistochemical expression profile for matrix metalloproteinase (MMP)-3, tissue inhibitor of metalloproteinases (TIMP)-1, and TIMP-2. (a) **Representative immunohistochemical loaded disc nucleus pulposus (NP) and annulus fibrosus (AF) sections for MMP-3, TIMP-1, TIMP-2, and negative control at 0, 7, 28, and 56 days of loading (bars: 50 μm). **(b) **Percentage of immunopositive cells of MMP-3 at 0, 7, 28, and 56 days in the loaded disc = NP: 44.1, 84.7, 79.0, 63.8, respectively, and AF: 19.5, 62.5, 60.4, 52.2, respectively; TIMP-1 = NP: 41.1, 36.7, 34.2, 9.4, respectively, and AF: 17.2, 20.8, 29.3, 11.9, respectively; TIMP-2 = NP: 47.2, 54.6, 27.6, 9.5, respectively, and AF: 15.8, 26.3, 25.3, 16.2, respectively. *P < 0.05 when compared between loaded and unloaded conditions. ^†^*P *< 0.05 when compared between different time points.

**Figure 4:**
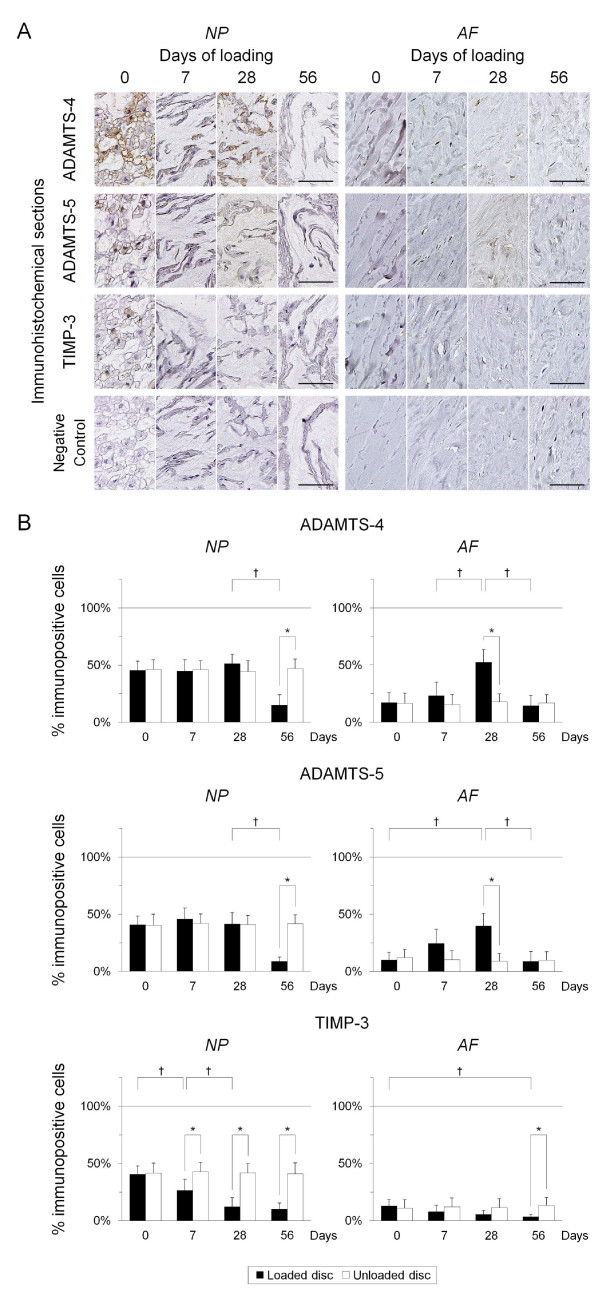
**Immunohistochemical expression profile for a disintegrin and metalloproteinase with thrombospondin motifs (ADAMTS)-4, ADAMTS-5, and tissue inhibitor of metalloproteinases (TIMP)-3. (a) **Representative immunohistochemical loaded disc nucleus pulposus (NP) and annulus fibrosus (AF) sections for ADAMTS-4, ADAMTS-5, TIMP-3, and negative control at 0, 7, 28, and 56 days of loading (bars: 50 μm). **(b) **Percentage of immunopositive cells of ADAMTS-4 at 0, 7, 28, and 56 days in the loaded disc = NP: 45.4, 44.7, 51.1, 14.9, respectively, and AF: 17.1, 23.0, 52.4, 14.5, respectively; ADAMTS-5 = NP: 40.6, 45.7, 41.5, 8.7, respectively, and AF: 9.9, 24.3, 39.7, 8.8, respectively; TIMP-3 = NP: 40.5, 26.3, 12.1, 10.1, respectively, and AF: 13.0, 7.8, 5.5, 3.5, respectively. *P < 0.05 when compared between loaded and unloaded conditions. ^†^*P *< 0.05 when compared between different time points.

Immunoreactivity for all examined genes was predominantly localized in the cytoplasm of disc cells and higher in NP cells than AF cells; particularly, large NP cells with vacuoles, suspecting notochordal origin, showed strong immunoreactivity (Figures 3a and 4a). Immunopositivity for all studied genes was detected in sham and unloaded discs and also generally higher in the NP than the AF (Figures 3b and 4b). No immunopositivity was detected in the matrix. IgG controls were negative, and positive controls showed strong positive stainings.

The percentage of immunopositive cells of MMP-3 in the NP significantly increased from 7 to 56 days (*P *< 0.05) despite a slight decrease at 56 days. The percentage of immunopositive cells of MMP-3 in the AF significantly increased from seven days (*P *< 0.05) and retained the enhanced expression through 56 days. The percentage of immunopositive cells of TIMP-1 in the NP had no significant change through 28 days but significantly decreased at 56 days (*P *< 0.05). The percentage of immunopositive cells of TIMP-1 in the AF did not show any significant change over the loading duration. The percentage of immunopositive cells of TIMP-2 in the NP significantly decreased at 28 and 56 days (*P *< 0.05). The percentage of immunopositive cells of TIMP-2 in the AF did not show any significant change throughout (Figure 3b).

The percentage of immunopositive cells of ADAMTS-4 and ADAMTS-5 in the NP showed no significant change through 28 days but significantly decreased at 56 days (*P *< 0.05). The percentage of immunopositive cells of ADAMTS-4 and ADAMTS-5 in the AF exhibited a transient increase at 28 days (*P *< 0.05). The percentage of immunopositive cells of TIMP-3 in the NP, despite possessing detectable staining in 0-day non-degenerated discs, consistently decreased until levels were nearly undetectable (*P *< 0.05). The percentage of immunopositive cells of TIMP-3 in the AF remained low throughout the study period with a significant decrease at 56 days (*P *< 0.05) (Figure 4b).

#### Aggrecanolytic activity

**Figure 5:**
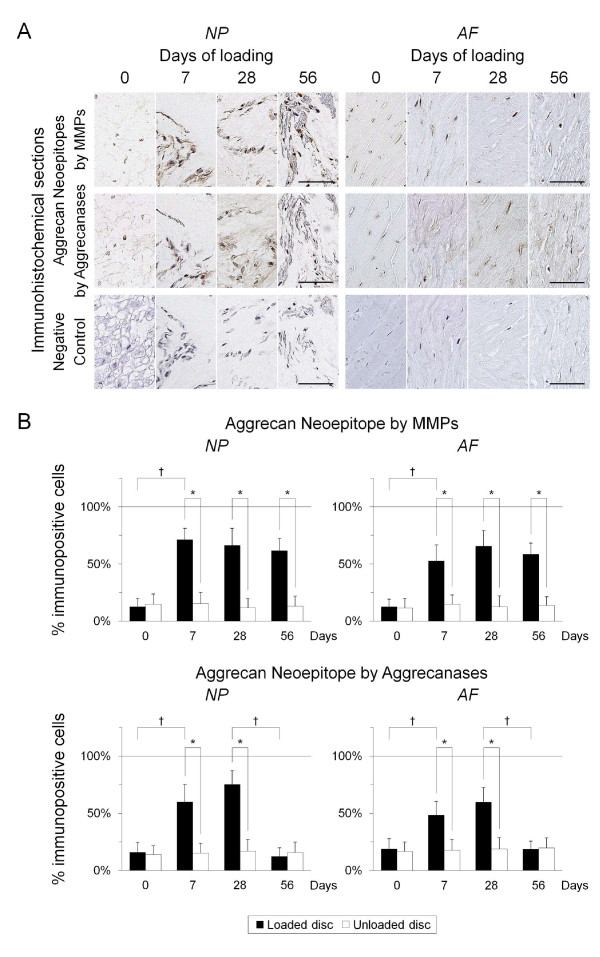
**Immunohistochemical expression profile for matrix metalloproteinase (MMP) and aggrecanase cleavage products of aggrecan. (a) **Representative immunohistochemical loaded disc nucleus pulposus (NP) and annulus fibrosus (AF) sections for aggrecan neoepitopes generated by MMPs and aggrecanases and negative control at 0, 7, 28, and 56 days of loading (bars: 50 μm). **(b) **Percentage of immunopositive cells of MMP-cleaved aggrecan neoepitope at 0, 7, 28, and 56 days in the loaded disc = NP: 12.6, 71.2, 66.3, 61.5, respectively, and AF: 12.6, 52.6, 65.6, 58.5, respectively; aggrecanase-cleaved aggrecan neoepitope = NP: 15.9, 59.9, 75.2, 12.3, respectively, and AF: 18.7, 48.3, 59.7, 18.4, respectively. *P < 0.05 when compared between loaded and unloaded conditions. ^†^*P *< 0.05 when compared between different time points.

Few positive stainings for MMP-generated and aggrecanase-generated aggrecan neoepitopes were detected in the 0-day NP and AF (Figure 5a). The percentage of immunopositive cells surrounded by MMP cleavage aggrecan neoepitope-positive matrix significantly increased from 7 to 56 days (*P *< 0.05) in the NP and AF. The percentage of immunopositive cells surrounded by aggrecanase cleavage aggrecan products significantly increased at 7 and 28 days (*P *< 0.05) but significantly decreased at 56 days (*P *< 0.05) in the NP and AF (Figure 5b).

## Discussion

Our results with the static compression model may be summarized in five major findings. First, real-time RT-PCR demonstrated up-regulated *MMPs*, lesser up-regulated *ADAMTSs*, and unchanged or down-regulated *TIMPs *during 56 days. Second, immnohistochemistry showed that the percentage of immunopositive cells of MMP-3 increased from 7 to 56 days while those of TIMP-1 and TIMP-2 were unchanged or decreased in the NP and AF. Third, in the NP, the percentage of immunopositive cells of ADAMTS-4 and ADAMTS-5 were unchanged through 28 days but decreased at 56 days while those of TIMP-3 consistently decreased from seven days. Fourth, in the AF, the percentage of immunopositive cells of ADAMTS-4 and ADAMTS-5 transiently increased at 28 days while those of TIMP-3 remained low throughout the study. Fifth, the percentage of immunopositive cells of MMP-cleaved aggrecan neoepitope increased from 7 to 56 days while those of aggrecanase-cleaved aggrecan neoepitope increased at 7 and 28 days but decreased at 56 days in the NP and AF. These findings indicate this rat tail sustained static compression model mimics ECM metabolic imbalances of MMPs, aggrecanases, and TIMPs in human degenerative discs. A dominant imbalance of MMP-3/TIMP-1 and TIMP-2 relative to ADAMTS-4 and ADAMTS-5/TIMP-3 signifies an advanced stage of intervertebral disc degeneration.

We previously reported that sustained static compression induced disc height loss in radiographs and lower NP intensity in T2-weighted MRIs [38]. Histological sections showed cell decrease with altered detectable phenotypes, scar formation, tissue defect, and a decrease of proteoglycans in Safranin-O staining [38,41]. In the current study, sustained static compression further induced *aggrecan-1 *and *collagen type 2-α1 *mRNA down-regulation and *collagen type 1-α1 *mRNA up-regulation (Figure 2c). These macro, micro, and biological findings have a good accordance with human degenerated discs [3,5,22,45] and other static compression models [25,28-30,34,35].

Our rat tail *MMP *mRNA up-regulation paralleled degeneration (Figure 2a), consistent with human disc findings [12-16,19]. A significant correlation between elevated MMP-2 and MMP-9 levels and degenerative disc grades was reported by Crean and colleagues [13]. Weiler and colleagues found that MMP-1, MMP-2, and MMP-3 expression highly correlated with cleft and scar formation in degenerated discs [15]. The gene expression study by Bachmeier and colleagues revealed that MMP-3 up-regulation highly depended on histological evidence of disc degeneration [19]. Furthermore, genomic anomalies of MMPs have significant involvement in disc degeneration [46-49]. A polymorphism in the promoter of MMP-1 [48], MMP-2 [47], MMP-3 [46], and MMP-9 [49] genes, which enhance promoter activity, is associated with a higher prevalence of lumbar disc degeneration in Japanese elderly patients [46] and Chinese adult cohorts [47-49]. Thus, the progression of disc degeneration strongly correlates with MMP up-regulation. The static compression model simulates MMP expression in intervertebral disc degeneration.

Our rat tail *ADAMTS-4 *mRNA up-regulation accompanied degeneration (Figure 2a), consistent with human discs [16-18]. On the other hand, our rat tail model exhibited no significant mRNA change of *ADAMTS-5 *(Figure 2a). ADAMTS-5 expression is controversial in human discs [17,18]. Pockert and colleagues reported ADAMTS-5 up-regulation correlated with histological grades of degenerative discs [18] whereas Patel and colleagues noted ADAMTS-5 expression level did not differ between histological grades [17]. In osteoarthritis cartilage, ADAMTS-5 is thought to have a more influential role than ADAMTS-4 [50,51]. ADAMTS-5 thus has the potential to play a role in intervertebral disc degeneration; however, further investigations are needed.

Our rat tail model demonstrated no consistent mRNA change of *TIMP-1 *and *TIMP-2 *and consistent mRNA down-regulation of *TIMP-3 *(Figure 2b). TIMP expression is also controversial in human discs [12,16,18,19]. Bachmeier and colleagues described *TIMP-1 *and *TIMP-2 *mRNA up-regulation in lumbar discs with degeneration [19]. In the investigation by Le Maitre and colleagues, up-regulation of TIMP-1 and TIMP-2, but not TIMP-3, correlated with the severity of histological degeneration [16]. No correlation of TIMP-3 with degeneration in the NP and inner AF and a negative correlation in the outer AF were observed by Pockert and colleagues [18]. Partially supporting these human data, the current animal model results indicate a differential expression pattern of TIMP-3 in disc degeneration. In ECM metabolism, much evidence for TIMP functions has been accumulated [7,8]; TIMPs inhibit MMPs by 1:1 interaction with zinc-binding site [8], TIMP-1 is the main inhibitor of MMP-3 [52], TIMP-2 inhibits MMP-2 [53] and MMP-14 [54], TIMP-3 is a potent inhibitor of ADAMTS-4 and ADAMTS-5 [55], and TIMP-1, TIMP-2, and TIMP-4 do not inhibit ADAMTS-4 [56]. Therefore, ratios of MMPs/TIMP-1 and TIMP-2 and ADAMTS-4 and ADAMTS-5/TIMP-3 must remain balanced to maintain matrix homeostasis. In human degenerative discs, it is difficult to profile these balances *in vivo*; however, the static compression model facilitates detailed longitudinal analysis of these balances in disc ECM metabolism during degeneration.

Our rat tail model showed mRNA up-regulation of *TNF-α *but not *ILs *(Figure 2d); however, human disc tissues frequently demonstrate up-regulation of TNF-α [57,58], IL-1α [59], IL-1β [58,59], and IL-6 [60]. In human non-herniated degenerative discs, large cleft formation and immunocompetent CD68-positive cells around cleft are observed [61]. In the repeated stab model, TNF-α, IL-1β, and IL-8 production in p38-positive cells is detected around the stab wound [62]. However, the static compression model does not present any large cleft formation or radial wound from the NP through the AF [28-30,38], potentially differing from physiological degeneration in the production mechanism of pro-inflammatory cytokines. TNF-α stimulates collagenase gene transcription by prolonged activation of *Jun *gene expression in fibroblasts [63]. TNF-α induces the production of nitric oxide [64], which can inhibit proteoglycan synthesis [65] and enhance MMP activity [66] in chondrocytes. TNF-α reduces the gene for Sox9 [67] which is required for the expression of chondrocyte-specific markers *aggrecan-1 *and *collagen type 2-α1 *[68]. Thus, *TNF-α *up-regulation in this model may indicate TNF-α contribution to the pathogenesis of disc degeneration.

To further characterize this cellular pathobiology, immunohistochemical study for MMPs, ADAMTSs, and TIMPs and their aggrecanolytic activity was performed. We investigated MMP-3 immunopositivity as representative of MMPs, judging from published evidence highlighting the significant importance of MMP-3 in disc degeneration [12,19]. MMP-3 is a major aggrecan-degrading MMP and activates many other pro-MMPs such as MMP-1, MMP-7, MMP-8, MMP-9, and MMP-13 [8]. In the NP, the prominent MMP-3/TIMP-1 and TIMP-2 imbalance (Figures 3a and 3b) with a persistent increase of MMP-cleaved aggrecan neoepitope (Figures 5a and 5b) was observed. Meanwhile, the modest ADAMTS-4 and ADAMTS-5/TIMP-3 imbalance (Figures 4a and 4b) with a transient increase of aggrecanase-cleaved aggrecan neoepitope (Figures 5a and 5b) was detected. Human disc studies have shown controversial findings of MMP-generated and aggrecanase-generated aggrecan fragments during degeneration [14,17,18,69]. Aggrecan neoepitopes cleaved by MMPs and aggrecanases are more frequently detected in degenerated than non-degenerated discs [18]. However, with advancing degeneration, the MMP-cleaved neoepitope abundance is constant in the NP and AF [69], whereas the aggrecanase-cleaved neoepitope abundance is unchanged [17] or decreased [69] in the NP but remains constant in the AF [17,69]. Aggrecanase-cleaved neoepitope is less abundant than MMP-cleaved neoepitope in degenerated discs [14]. The static compression model recapitulates these pieces of evidence regarding disc matrix aggrecan degradation; advancing degeneration does not necessarily imply increased aggrecan fragments, particularly by aggrecanase cleavage.

In this study, gene expression results with real-time RT-PCR and immunohistochemistry were partially mismatched. In the MMP-3/TIMP-1 and TIMP-2 imbalance, MMP-3 mRNA and protein up-regulation was consistent while TIMP-1 and TIMP-2 protein down-regulation at 28 to 56 days was inconsistent with their constant mRNA expression. These TIMP protein findings are not in agreement with those reported by Le Maitre and colleagues [16]; however, they do corroborate those by Kanemoto and colleagues where 78.1% of cervical spondylosis and 93.3% of lumbar spondylosis specimens were MMP-3 positive and TIMP-1 negative [12]. A prolonged catabolic shift might lead to decreased TIMP-1 and TIMP-2 protein. In the ADAMTS-4 and ADAMTS-5/TIMP-3 imbalance, TIMP-3 mRNA and protein down-regulation was consistent while no obvious change of ADAMTS-4 protein expression at 7 and 28 days was inconsistent with its mRNA up-regulation. Immunohistochemistry shows localization, but not productive quantity; therefore, it may be difficult to detect small expression change. This ADAMTS-4 finding possibly indicates that the ADAMTS-4 and ADAMTS-5/TIMP-3 imbalance is primarily due to TIMP-3 down-regulation in the NP. ADAMTS-4 and ADAMTS-5 protein down-regulation at 56 days was further inconsistent with their elevated or constant mRNA expression. These ADAMTS findings possibly correlate with decreased aggrecanase-cleaved aggrecan neoepitope. The observed discrepancy between mRNA and protein expression in the NP might be explained by prolonged, prominent MMP-induced aggrecanolysis and shortened, modest aggrecanase-induced aggrecanolysis; however, further investigations are required to understand the regulation mechanism of MMPs, ADAMTSs, and TIMPs at the post-transcriptional level.

The pathomechanism of AF degradation has not been clarified in detail. The annulus matrix comprises collagens, proteoglycans, and elastic fibers -- elastin and microfibril such as glycoprotein fibrillins [70]. Elastin is readily degraded by MMP-2, MMP-3, MMP-7, MMP-9, MMP-10, and MMP-12 [8,71]. Fibrillins are degraded by MMP-2, MMP-3, MMP-9, MMP-12, MMP-13, and MMP-14 [72]. This study lacked real-time RT-PCR analysis of AF tissue, which is an inherent limitation. In immunohistochemistry, the prominent MMP-3/TIMP-1 and TIMP-2 imbalance (Figures 3a and 3b) with a persistent increase of MMP-cleaved aggrecan neoepitope (Figures 5a and 5b) was observed in the AF as well as in the NP. Meanwhile, the ADAMTS-4 and ADAMTS-5/TIMP-3 imbalance in the AF had a pattern different from that in the NP; more remarkable ADAMTS-4 and ADAMTS-5 up-regulation than TIMP-3 down-regulation was detected (Figures 4a and 4b). This imbalance appeared to produce a transient increase of aggrecanase-cleaved aggrecan neoepitope at much the same time as the NP (Figures 5a and 5b). The histomorphological study by Boos and colleagues showed the NP was more severely degenerated in the same age group than the AF [22]; however, our biological findings indicate AF degeneration occurs simultaneously with NP degeneration.

**Figure 6:**
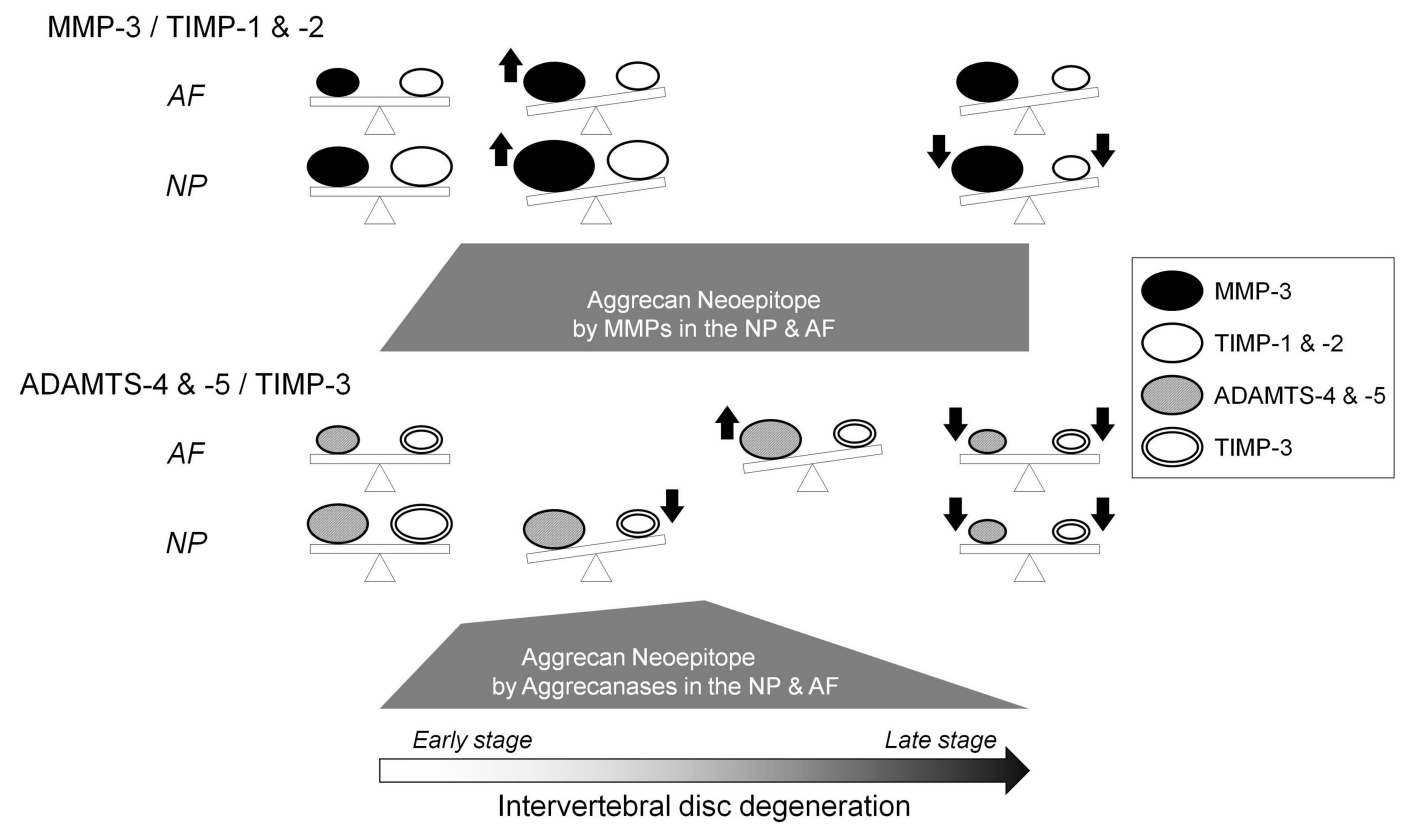
**Schematic illustration summarizing static compression loading-induced extracellular matrix metabolic imbalances of matrix metalloproteinases (MMPs), aggrecanases belonging to a disintegrin and metalloproteinase with thrombospondin motifs (ADAMTS) family, and tissue inhibitors of metalloproteinases (TIMPs) in the disc nucleus pulposus (NP) and annulus fibrosus (AF)**. A dominant imbalance of MMP-3/TIMP-1 and TIMP-2 relative to ADAMTS-4 and ADAMTS-5/TIMP-3 is an indication of an advanced stage of intervertebral disc degeneration.

Our rat tail immunohistochemical results are summarized in Figure 6. Both imbalances of MMP-3/TIMP-1 and TIMP-2 and ADAMTS-4 and ADAMTS-5/TIMP-3 work in the early to middle stages of 7 and 28 days; however, the MMP-3/TIMP-1 and TIMP-2 imbalance is more severe than the ADAMTS-4 and ADAMTS-5/TIMP-3 imbalance at the late stage of 56 days. This provides a sound argument for low aggrecanase activity in the discs with advanced degeneration. In ECM metabolism, the relative importance of MMPs and aggrecanases has long been debated. Little and colleagues reported catabolic aggrecan degradation in normal and osteoarthritis cartilage primarily involved cleavage by aggrecanase and not by MMPs [73]. It was found by Karsdal and colleagues that MMP-mediated degradation of aggrecan and collagen type 2 caused irreversible damage for cartilage, while aggrecanase-mediated degradation of aggrecan was fully reversible [74]. Integrated with these reports, our findings show that a state of dominant MMP-3/TIMP-1 and TIMP-2 imbalance relative to ADAMTS-4 and ADAMTS-5/TIMP-3 imbalance may indicate an irreversible stage of intervertebral disc degeneration.

This rodent disc study provides information not only about staged processes involved in disc degeneration but also about its possible pathogenesis. In particular, the loss of NP notochordal cells induced by sustained static compression should be noted. In our previous study, immunofluorescence exhibited that the number of NP cells decreased to 43.0% during 56 days of loading [41]. In the *in vivo *study by Guehring and colleagues, sustained static compression induced more remarkable decrease of notochordal cells than of chondrocyte-like cells with a total cell decrease of more than 50% over 56 days [75]. Severity of cell decrease is similar in both studies; although we did not identify notochordal cells using immunostaining for the markers, it is speculated that our rat tail disc significantly loses notochordal cells following compression. In seven-day loaded disc sections, we observed an apparent decrease of large vacuolated cells suspecting notochordal origin, supporting the proposal by Guehring and colleagues that notochordal cells are less resistant to mechanical stress than chondrocyte-like cells [75].

We further found more distinct immunoreactivity for MMP, ADAMTS, and TIMP enzymes in notochordal cells than in chodrocyte-like cells (Figures 3a and 4a). In 0-day discs, large vacuolated NP cells showed strong immunoreactivity despite little detection of aggrecan fragments. In seven-day loaded discs, many NP cells were chondrocyte-like and demonstrated generally weaker immunoreactivity with increased detection of aggrecan neoepitopes, which was irrespective of immunopositivity affected by loading. This finding gives rise to the possibility that notochordal cells play an important role in matrix homeostasis, providing a plausible explanation for the observed higher baseline expression levels of MMPs, ADAMTSs, and TIMPs in the NP than in the AF and differential imbalanced pattern of ADAMTS-4 and ADAMTS-5/TIMP-3 between the NP and AF. Notochordal cells produce a larger amount of proteoglycans than chondrocyte-like cells [76,77] and stimulate chondrocyte-like cells to produce proteoglycans [78,79]. Proteoglycan loss is an early, significant biochemical change to occur in disc degeneration [3,4]. Human disc NP tissues have a higher content of glycosaminoglycans than AF in donors aged 25 years or younger but thereafter lose them; further, NP specimens have a higher level of newly synthesized aggrecan than AF in donors five years or younger however lose it markedly by 5 to 15 years [5], corresponding well with the disappearance of notochordal cells [22]. In keeping with these findings, our study results indicate that the loss of notochordal cells is potentially linked with the initiation of disc degeneration.

A major limitation of this study is that the rat tail static compression model does not perfectly reflect all aspects of human disc degeneration. This model's unphysiological situation -- immobility, extended pressure, and absence of trauma -- could lead to some contradictions to humans, such as lack of radial tears, mucous degeneration, and granular changes in histology and little involvement of inflammation. It could also explain the observed simultaneous NP and AF degeneration.

Further studies using the static compression model should be conducted to detect the turning point from reversible to irreversible degeneration, providing key information to prevent degenerative disc diseases.

## Conclusions

This rat tail sustained static compression model mimics ECM metabolic imbalances of MMPs, aggrecanases, and TIMPs in human intervertebral disc degeneration. In early stages, MMP-3/TIMP-1 and TIMP-2 and ADAMTS-4 and ADAMTS-5/TIMP-3 imbalances exist. In the late stage, the MMP-3/TIMP-1 and TIMP-2 imbalance dominates. These imbalances and their effects on aggrecanolysis are common in the NP and AF. A dominant imbalance of MMP-3/TIMP-1 and TIMP-2 relative to ADAMTS-4 and ADAMTS-5/TIMP-3 is a possible indication of an advanced, irreversible stage of intervertebral disc degeneration.

## Abbreviations

ADAMTS: a disintegrin and metalloproteinase with thrombospondin motifs; AF: annulus fibrosus; ANOVA: analysis of variance; C: coccygeal; ECM: extracellular matrix; GAPDH: glyceraldehyde 3-phosphate dehydrogenase; IL: interleukin; MMP: matrix metalloproteinase; MRI: magnetic resonance imaging; mRNA: messenger RNA; NP: nucleus pulposus; PCR: polymerase chain reaction; RT: reverse transcription; TIMP: tissue inhibitor of metalloproteinases; TNF: tumor necrosis factor.

## Competing interests

The authors declare that they have no competing interests.

## Authors' contributions

TY conceived the study, secured funding, participated in its conception and design, acquisition of data, and analysis and interpretation of data, and drafted the manuscript. TT participated in analysis and interpretation of data and helped to draft the manuscript. TS participated in acquisition of data and analysis and interpretation of data. KK participated in analysis and interpretation of data. KM also participated in analysis and interpretation of data. MD conceived the study, secured funding, and participated in its conception and design and analysis and interpretation of data. MK helped to secure funding and participated in analysis and interpretation of data. KN conceived the study, secured funding, participated in its conception and design and analysis and interpretation of data, and helped to draft the manuscript. All authors read and approved the final manuscript.

## References

[B1] Andersson GB Epidemiological features of chronic low-back pain Lancet 1999 354 581 585 10.1016/S0140-6736(99)01312-4 10470716

[B2] Luoma K Riihimaki H Luukkonen R Raininko R Viikari-Juntura E Lamminen A Low back pain in relation to lumbar disc degeneration Spine (Phila Pa 1976) 2000 25 487 492 10.1097/00007632-200002150-00016 10707396

[B3] Lyons G Eisenstein SM Sweet MB Biochemical changes in intervertebral disc degeneration Biochim Biophys Acta 1981 673 443 453 7225426 10.1016/0304-4165(81)90476-1

[B4] Pearce RH Grimmer BJ Target tissue models: the proteoglycans and degeneration of the human intervertebral disc J Rheumatol Suppl 1983 11 108 110 6583410

[B5] Antoniou J Steffen T Nelson F Winterbottom N Hollander AP Poole RA Aebi M Alini M The human lumbar intervertebral disc: evidence for changes in the biosynthesis and denaturation of the extracellular matrix with growth, maturation, ageing, and degeneration J Clin Invest 1996 98 996 1003 10.1172/JCI118884 8770872 PMC507515

[B6] Eyre DR Muir H Types I and II collagens in intervertebral disc. Interchanging radial distributions in annulus fibrosus Biochem J 1976 157 267 270 962859 10.1042/bj1570267 PMC1163842

[B7] Nagase H Kashiwagi M Aggrecanases and cartilage matrix degradation Arthritis Res Ther 2003 5 94 103 12718749 10.1186/ar630 PMC165039

[B8] Visse R Nagase H Matrix metalloproteinases and tissue inhibitors of metalloproteinases: structure, function, and biochemistry Circ Res 2003 92 827 839 10.1161/01.RES.0000070112.80711.3D 12730128

[B9] Yoshihara Y Nakamura H Obata K Yamada H Hayakawa T Fujikawa K Okada Y Matrix metalloproteinases and tissue inhibitors of metalloproteinases in synovial fluids from patients with rheumatoid arthritis or osteoarthritis Ann Rheum Dis 2000 59 455 461 10.1136/ard.59.6.455 10834863 PMC1753174

[B10] Yamanishi Y Boyle DL Clark M Maki RA Tortorella MD Arner EC Firestein GS Expression and regulation of aggrecanase in arthritis: the role of TGF-beta J Immunol 2002 168 1405 1412 11801682 10.4049/jimmunol.168.3.1405

[B11] Davidson RK Waters JG Kevorkian L Darrah C Cooper A Donell ST Clark IM Expression profiling of metalloproteinases and their inhibitors in synovium and cartilage Arthritis Res Ther 2006 8 R124 10.1186/ar2013 16859525 PMC1779413

[B12] Kanemoto M Hukuda S Komiya Y Katsuura A Nishioka J Immunohistochemical study of matrix metalloproteinase-3 and tissue inhibitor of metalloproteinase-1 human intervertebral discs Spine (Phila Pa 1976) 1996 21 1 8 10.1097/00007632-199601010-00001 9122749

[B13] Crean JK Roberts S Jaffray DC Eisenstein SM Duance VC Matrix metalloproteinases in the human intervertebral disc: role in disc degeneration and scoliosis Spine (Phila Pa 1976) 1997 22 2877 2884 10.1097/00007632-199712150-00010 9431623

[B14] Roberts S Caterson B Menage J Evans EH Jaffray DC Eisenstein SM Matrix metalloproteinases and aggrecanase: their role in disorders of the human intervertebral disc Spine (Phila Pa 1976) 2000 25 3005 3013 10.1097/00007632-200012010-00007 11145811

[B15] Weiler C Nerlich AG Zipperer J Bachmeier BE Boos N 2002 SSE Award Competition in Basic Science: expression of major matrix metalloproteinases is associated with intervertebral disc degradation and resorption Eur Spine J 2002 11 308 320 10.1007/s00586-002-0472-0 12193991 PMC3610483

[B16] Le Maitre CL Freemont AJ Hoyland JA Localization of degradative enzymes and their inhibitors in the degenerate human intervertebral disc J Pathol 2004 204 47 54 10.1002/path.1608 15307137

[B17] Patel KP Sandy JD Akeda K Miyamoto K Chujo T An HS Masuda K Aggrecanases and aggrecanase-generated fragments in the human intervertebral disc at early and advanced stages of disc degeneration Spine (Phila Pa 1976) 2007 32 2596 2603 10.1097/BRS.0b013e318158cb85 17978660

[B18] Pockert AJ Richardson SM Le Maitre CL Lyon M Deakin JA Buttle DJ Freemont AJ Hoyland JA Modified expression of the ADAMTS enzymes and tissue inhibitor of metalloproteinases 3 during human intervertebral disc degeneration Arthritis Rheum 2009 60 482 491 10.1002/art.24291 19180493

[B19] Bachmeier BE Nerlich A Mittermaier N Weiler C Lumenta C Wuertz K Boos N Matrix metalloproteinase expression levels suggest distinct enzyme roles during lumbar disc herniation and degeneration Eur Spine J 2009 18 1573 1586 10.1007/s00586-009-1031-8 19466462 PMC2899407

[B20] Urban JP Smith S Fairbank JC Nutrition of the intervertebral disc Spine (Phila Pa 1976) 2004 29 2700 2709 10.1097/01.brs.0000146499.97948.52 15564919

[B21] Alini M Eisenstein SM Ito K Little C Kettler AA Masuda K Melrose J Ralphs J Stokes I Wilke HJ Are animal models useful for studying human disc disorders/degeneration? Eur Spine J 2008 17 2 19 10.1007/s00586-007-0414-y 17632738 PMC2365516

[B22] Boos N Weissbach S Rohrbach H Weiler C Spratt KF Nerlich AG Classification of age-related changes in lumbar intervertebral discs: 2002 Volvo Award in basic science Spine (Phila Pa 1976) 2002 27 2631 2644 10.1097/00007632-200212010-00002 12461389

[B23] Oegema TR Jr The role of disc cell heterogeneity in determining disc biochemistry: a speculation Biochem Soc Trans 2002 30 839 844 12440929 10.1042/bst0300839

[B24] Hunter CJ Matyas JR Duncan NA The notochordal cell in the nucleus pulposus: a review in the context of tissue engineering Tissue Eng 2003 9 667 677 10.1089/107632703768247368 13678445

[B25] Ching CT Chow DH Yao FY Holmes AD Changes in nuclear composition following cyclic compression of the intervertebral disc in an in vivo rat-tail model Med Eng Phys 2004 26 587 594 10.1016/j.medengphy.2004.03.006 15271286

[B26] Masuoka K Michalek AJ MacLean JJ Stokes IA Iatridis JC Different effects of static versus cyclic compressive loading on rat intervertebral disc height and water loss in vitro Spine (Phila Pa 1976) 2007 32 1974 1979 10.1097/BRS.0b013e318133d591 17700443 PMC2570779

[B27] Wang DL Jiang SD Dai LY Biologic response of the intervertebral disc to static and dynamic compression in vitro Spine (Phila Pa 1976) 2007 32 2521 2528 10.1097/BRS.0b013e318158cb61 17978649

[B28] Lotz JC Colliou OK Chin JR Duncan NA Liebenberg E Compression-induced degeneration of the intervertebral disc: an in vivo mouse model and finite-element study Spine (Phila Pa 1976) 1998 23 2493 2506 10.1097/00007632-199812010-00004 9854748

[B29] Hutton WC Ganey TM Elmer WA Kozlowska E Ugbo JL Doh ES Whitesides TE Jr Does long-term compressive loading on the intervertebral disc cause degeneration? Spine (Phila Pa 1976) 2000 25 2993 3004 10.1097/00007632-200012010-00006 11145810

[B30] Kroeber MW Unglaub F Wang H Schmid C Thomsen M Nerlich A Richter W New in vivo animal model to create intervertebral disc degeneration and to investigate the effects of therapeutic strategies to stimulate disc regeneration Spine (Phila Pa 1976) 2002 27 2684 2690 10.1097/00007632-200212010-00007 12461394

[B31] Lotz JC Chin JR Intervertebral disc cell death is dependent on the magnitude and duration of spinal loading Spine (Phila Pa 1976) 2000 25 1477 1483 10.1097/00007632-200006150-00005 10851095

[B32] Rannou F Lee TS Zhou RH Chin J Lotz JC Mayoux-Benhamou MA Barbet JP Chevrot A Shyy JY Intervertebral disc degeneration: the role of the mitochondrial pathway in annulus fibrosus cell apoptosis induced by overload Am J Pathol 2004 164 915 924 10.1016/S0002-9440(10)63179-3 14982845 PMC1613264

[B33] Iatridis JC Mente PL Stokes IA Aronsson DD Alini M Compression-induced changes in intervertebral disc properties in a rat tail model Spine (Phila Pa 1976) 1999 24 996 1002 10.1097/00007632-199905150-00013 10332792

[B34] Guehring T Omlor GW Lorenz H Bertram H Steck E Richter W Carstens C Kroeber M Stimulation of gene expression and loss of anular architecture caused by experimental disc degeneration--an in vivo animal study Spine (Phila Pa 1976) 2005 30 2510 2515 10.1097/01.brs.0000186591.17114.e9 16284588

[B35] Omlor GW Lorenz H Engelleiter K Richter W Carstens C Kroeber MW Guehring T Changes in gene expression and protein distribution at different stages of mechanically induced disc degeneration--an in vivo study on the New Zealand white rabbit J Orthop Res 2006 24 385 392 10.1002/jor.20055 16479572

[B36] Wuertz K Godburn K MacLean JJ Barbir A Donnelly JS Roughley PJ Alini M Iatridis JC In vivo remodeling of intervertebral discs in response to short- and long-term dynamic compression J Orthop Res 2009 27 1235 1242 10.1002/jor.20867 19274755 PMC2757138

[B37] Hsieh AH Lotz JC Prolonged spinal loading induces matrix metalloproteinase-2 activation in intervertebral discs Spine (Phila Pa 1976) 2003 28 1781 1788 10.1097/01.BRS.0000083282.82244.F3 12923463

[B38] Yurube T Nishida K Suzuki T Kaneyama S Zhang Z Kakutani K Maeno K Takada T Fujii M Kurosaka M Doita M Matrix metalloproteinase (MMP)-3 gene up-regulation in a rat tail compression loading-induced disc degeneration model J Orthop Res 2010 28 1026 1032 20162718 10.1002/jor.21116

[B39] Hughes PC Tanner JM The assessment of skeletal maturity in the growing rat J Anat 1970 106 371 402 4315144 PMC1233709

[B40] Elliott DM Sarver JJ Young investigator award winner: validation of the mouse and rat disc as mechanical models of the human lumbar disc Spine (Phila Pa 1976) 2004 29 713 722 10.1097/01.BRS.0000116982.19331.EA 15087791

[B41] Yurube T Takada T Hirata H Kakutani K Maeno K Zhang Z Yamamoto J Doita M Kurosaka M Nishida K Modified house-keeping gene expression in a rat tail compression loading-induced disc degeneration model J Orthop Res 2011 29 1284 1290 10.1002/jor.21406 21387398

[B42] Balbin M Fueyo A Knauper V Lopez JM Alvarez J Sanchez LM Quesada V Bordallo J Murphy G Lopez-Otin C Identification and enzymatic characterization of two diverging murine counterparts of human interstitial collagenase (MMP-1) expressed at sites of embryo implantation J Biol Chem 2001 276 10253 10262 10.1074/jbc.M009586200 11113146

[B43] Vincenti MP Coon CI Mengshol JA Yocum S Mitchell P Brinckerhoff CE Cloning of the gene for interstitial collagenase-3 (matrix metalloproteinase-13) from rabbit synovial fibroblasts: differential expression with collagenase-1 (matrix metalloproteinase-1) Biochem J 1998 331 Pt 1 341 346 9512498 10.1042/bj3310341 PMC1219357

[B44] Livak KJ Schmittgen TD Analysis of relative gene expression data using real-time quantitative PCR and the 2(-Delta Delta C(T)) Method Methods 2001 25 402 408 10.1006/meth.2001.1262 11846609

[B45] Benneker LM Heini PF Anderson SE Alini M Ito K Correlation of radiographic and MRI parameters to morphological and biochemical assessment of intervertebral disc degeneration Eur Spine J 2005 14 27 35 10.1007/s00586-004-0759-4 15723249 PMC3476685

[B46] Takahashi M Haro H Wakabayashi Y Kawa-uchi T Komori H Shinomiya K The association of degeneration of the intervertebral disc with 5a/6a polymorphism in the promoter of the human matrix metalloproteinase-3 gene J Bone Joint Surg Br 2001 83 491 495 10.1302/0301-620X.83B4.11617 11380116

[B47] Dong DM Yao M Liu B Sun CY Jiang YQ Wang YS Association between the -1306C/T polymorphism of matrix metalloproteinase-2 gene and lumbar disc disease in Chinese young adults Eur Spine J 2007 16 1958 1961 10.1007/s00586-007-0454-3 17680282 PMC2223336

[B48] Song YQ Ho DW Karppinen J Kao PY Fan BJ Luk KD Yip SP Leong JC Cheah KS Sham P Chan D Cheung KM Association between promoter -1607 polymorphism of MMP1 and lumbar disc disease in Southern Chinese BMC Med Genet 2008 9 38 18439317 10.1186/1471-2350-9-38 PMC2386444

[B49] Sun ZM Miao L Zhang YG Ming L Association between the -1562 C/T polymorphism of matrix metalloproteinase-9 gene and lumbar disc disease in the young adult population in North China Connect Tissue Res 2009 50 181 185 10.1080/03008200802585630 19444758

[B50] Glasson SS Askew R Sheppard B Carito B Blanchet T Ma HL Flannery CR Peluso D Kanki K Yang Z Majumdar MK Morris EA Deletion of active ADAMTS5 prevents cartilage degradation in a murine model of osteoarthritis Nature 2005 434 644 648 10.1038/nature03369 15800624

[B51] Stanton H Rogerson FM East CJ Golub SB Lawlor KE Meeker CT Little CB Last K Farmer PJ Campbell IK Fourie AM Fosang AJ ADAMTS5 is the major aggrecanase in mouse cartilage in vivo and in vitro Nature 2005 434 648 652 10.1038/nature03417 15800625

[B52] Gomis-Ruth FX Maskos K Betz M Bergner A Huber R Suzuki K Yoshida N Nagase H Brew K Bourenkov GP Bartunik H Bode W Mechanism of inhibition of the human matrix metalloproteinase stromelysin-1 by TIMP-1 Nature 1997 389 77 81 10.1038/37995 9288970

[B53] Morgunova E Tuuttila A Bergmann U Tryggvason K Structural insight into the complex formation of latent matrix metalloproteinase 2 with tissue inhibitor of metalloproteinase 2 Proc Natl Acad Sci USA 2002 99 7414 7419 10.1073/pnas.102185399 12032297 PMC124245

[B54] Zucker S Drews M Conner C Foda HD DeClerck YA Langley KE Bahou WF Docherty AJ Cao J Tissue inhibitor of metalloproteinase-2 (TIMP-2) binds to the catalytic domain of the cell surface receptor, membrane type 1-matrix metalloproteinase 1 (MT1-MMP) J Biol Chem 1998 273 1216 1222 10.1074/jbc.273.2.1216 9422789

[B55] Kashiwagi M Tortorella M Nagase H Brew K TIMP-3 is a potent inhibitor of aggrecanase 1 (ADAM-TS4) and aggrecanase 2 (ADAM-TS5) J Biol Chem 2001 276 12501 12504 10.1074/jbc.C000848200 11278243

[B56] Hashimoto G Aoki T Nakamura H Tanzawa K Okada Y Inhibition of ADAMTS4 (aggrecanase-1) by tissue inhibitors of metalloproteinases (TIMP-1, 2, 3 and 4) FEBS Lett 2001 494 192 195 10.1016/S0014-5793(01)02323-7 11311239

[B57] Weiler C Nerlich AG Bachmeier BE Boos N Expression and distribution of tumor necrosis factor alpha in human lumbar intervertebral discs: a study in surgical specimen and autopsy controls Spine (Phila Pa 1976) 2005 30 44 53 10.1097/01.brs.0000174529.07959.c0 15626980

[B58] Le Maitre CL Hoyland JA Freemont AJ Catabolic cytokine expression in degenerate and herniated human intervertebral discs: IL-1beta and TNFalpha expression profile Arthritis Res Ther 2007 9 R77 10.1186/ar2275 17688691 PMC2206382

[B59] Le Maitre CL Freemont AJ Hoyland JA The role of interleukin-1 in the pathogenesis of human intervertebral disc degeneration Arthritis Res Ther 2005 7 R732 745 10.1186/ar1732 15987475 PMC1175026

[B60] Burke JG Watson RW McCormack D Dowling FE Walsh MG Fitzpatrick JM Intervertebral discs which cause low back pain secrete high levels of proinflammatory mediators J Bone Joint Surg Br 2002 84 196 201 10.1302/0301-620X.84B2.12511 11924650

[B61] Nerlich AG Weiler C Zipperer J Narozny M Boos N Immunolocalization of phagocytic cells in normal and degenerated intervertebral discs Spine (Phila Pa 1976) 2002 27 2484 2490 10.1097/00007632-200211150-00012 12435979

[B62] Ulrich JA Liebenberg EC Thuillier DU Lotz JC ISSLS prize winner: repeated disc injury causes persistent inflammation Spine (Phila Pa 1976) 2007 32 2812 2819 10.1097/BRS.0b013e31815b9850 18246002

[B63] Brenner DA O'Hara M Angel P Chojkier M Karin M Prolonged activation of jun and collagenase genes by tumour necrosis factor-alpha Nature 1989 337 661 663 10.1038/337661a0 2537468

[B64] Palmer RM Hickery MS Charles IG Moncada S Bayliss MT Induction of nitric oxide synthase in human chondrocytes Biochem Biophys Res Commun 1993 193 398 405 10.1006/bbrc.1993.1637 7684906

[B65] Taskiran D Stefanovic-Racic M Georgescu H Evans C Nitric oxide mediates suppression of cartilage proteoglycan synthesis by interleukin-1 Biochem Biophys Res Commun 1994 200 142 148 10.1006/bbrc.1994.1426 7513156

[B66] Sasaki K Hattori T Fujisawa T Takahashi K Inoue H Takigawa M Nitric oxide mediates interleukin-1-induced gene expression of matrix metalloproteinases and basic fibroblast growth factor in cultured rabbit articular chondrocytes J Biochem 1998 123 431 439 9538225 10.1093/oxfordjournals.jbchem.a021955

[B67] Murakami S Lefebvre V de Crombrugghe B Potent inhibition of the master chondrogenic factor Sox9 gene by interleukin-1 and tumor necrosis factor-alpha J Biol Chem 2000 275 3687 3692 10.1074/jbc.275.5.3687 10652367

[B68] Bi W Deng JM Zhang Z Behringer RR de Crombrugghe B Sox9 is required for cartilage formation Nat Genet 1999 22 85 89 10.1038/8792 10319868

[B69] Sztrolovics R Alini M Roughley PJ Mort JS Aggrecan degradation in human intervertebral disc and articular cartilage Biochem J 1997 326 Pt 1 235 241 9337874 10.1042/bj3260235 PMC1218660

[B70] Smith LJ Fazzalari NL The elastic fibre network of the human lumbar anulus fibrosus: architecture, mechanical function and potential role in the progression of intervertebral disc degeneration Eur Spine J 2009 18 439 448 10.1007/s00586-009-0918-8 19263091 PMC2899476

[B71] Mecham RP Broekelmann TJ Fliszar CJ Shapiro SD Welgus HG Senior RM Elastin degradation by matrix metalloproteinases. Cleavage site specificity and mechanisms of elastolysis J Biol Chem 1997 272 18071 18076 10.1074/jbc.272.29.18071 9218437

[B72] Ashworth JL Murphy G Rock MJ Sherratt MJ Shapiro SD Shuttleworth CA Kielty CM Fibrillin degradation by matrix metalloproteinases: implications for connective tissue remodelling Biochem J 1999 340 Pt 1 171 181 10229672 PMC1220235

[B73] Little CB Flannery CR Hughes CE Mort JS Roughley PJ Dent C Caterson B Aggrecanase versus matrix metalloproteinases in the catabolism of the interglobular domain of aggrecan in vitro Biochem J 1999 344 Pt 1 61 68 10548534 PMC1220614

[B74] Karsdal MA Madsen SH Christiansen C Henriksen K Fosang AJ Sondergaard BC Cartilage degradation is fully reversible in the presence of aggrecanase but not matrix metalloproteinase activity Arthritis Res Ther 2008 10 R63 10.1186/ar2434 18513402 PMC2483454

[B75] Guehring T Nerlich A Kroeber M Richter W Omlor GW Sensitivity of notochordal disc cells to mechanical loading: an experimental animal study Eur Spine J 2010 19 113 121 10.1007/s00586-009-1217-0 19936803 PMC2899741

[B76] Cappello R Bird JL Pfeiffer D Bayliss MT Dudhia J Notochordal cell produce and assemble extracellular matrix in a distinct manner, which may be responsible for the maintenance of healthy nucleus pulposus Spine (Phila Pa 1976) 2006 31 873 882 10.1097/01.brs.0000209302.00820.fd 16622374

[B77] Miyazaki T Kobayashi S Takeno K Meir A Urban J Baba H A phenotypic comparison of proteoglycan production of intervertebral disc cells isolated from rats, rabbits, and bovine tails; which animal model is most suitable to study tissue engineering and biological repair of human disc disorders? Tissue Eng Part A 2009 15 3835 3846 10.1089/ten.tea.2009.0250 19681728

[B78] Aguiar DJ Johnson SL Oegema TR Notochordal cells interact with nucleus pulposus cells: regulation of proteoglycan synthesis Exp Cell Res 1999 246 129 137 10.1006/excr.1998.4287 9882522

[B79] Erwin WM Inman RD Notochord cells regulate intervertebral disc chondrocyte proteoglycan production and cell proliferation Spine (Phila Pa 1976) 2006 31 1094 1099 10.1097/01.brs.0000216593.97157.dd 16648742

